# A Regenerative Antioxidant Protocol of Vitamin E and ***α***-Lipoic Acid Ameliorates Cardiovascular and Metabolic Changes in Fructose-Fed Rats

**DOI:** 10.1155/2011/120801

**Published:** 2011-03-09

**Authors:** Jatin Patel, Nur Azim Matnor, Abishek Iyer, Lindsay Brown

**Affiliations:** ^1^School of Biomedical Sciences, The University of Queensland, Brisbane, QLD 4072, Australia; ^2^Department of Biological and Physical Sciences, University of Southern Queensland, Toowoomba, QLD 4350, Australia

## Abstract

Type 2 diabetes is a major cause of cardiovascular disease. We have determined whether the metabolic and cardiovascular changes induced by a diet high in fructose in young adult male Wistar rats could be prevented or reversed by chronic intervention with natural antioxidants. We administered a regenerative antioxidant protocol using two natural compounds: *α*-lipoic acid together with vitamin E (*α*-tocopherol alone or a tocotrienol-rich fraction), given as either a prevention or reversal protocol in the food. These rats developed glucose intolerance, hypertension, and increased collagen deposition in the heart together with an increased ventricular stiffness. Treatment with a fixed combination of vitamin E (either *α*-tocopherol or tocotrienol-rich fraction, 0.84 g/kg food) and *α*-lipoic acid (1.6 g/kg food) normalized glucose tolerance, blood pressure, cardiac collagen deposition, and ventricular stiffness in both prevention and reversal protocols in these fructose-fed rats. These results suggest that adequate antioxidant therapy can both prevent and reverse the metabolic and cardiovascular damage in type 2 diabetes.

## 1. Introduction

The epidemic of type 2 diabetes now affects approximately 150 million people worldwide, with a projected incidence of 300 million by the year 2025 [1, 2]. This epidemic has been attributed to high fat/high sugar intakes in modern diets, correlating with the increased use of fructose as a sweetener [1]. Dietary fructose undergoes rapid metabolism by the liver, causing changes in carbohydrate and lipid metabolism and hepatic inflammation leading to the development of hyperglycemia, insulin resistance, hyperinsulinemia, and hypertriglyceridemia as major risk factors for diabetic complications [2–4]. Rats fed a high fructose diet mimic the progression of type 2 diabetes seen in humans including glucose intolerance, increased oxidative stress, hypertension, and reduced myocardial and vascular compliance [5–9]. Fructose feeding initiates an increased mitochondrial formation of reactive oxygen species and, as a consequence, oxidative stress [10], producing hypertension and decreased myocardial compliance, as evidenced by improvements in symptoms following antioxidant therapy [11–13].

Naturally occurring antioxidants include vitamin E, a family of naturally occurring tocopherols and tocotrienols [14]. While many studies suggest that antioxidant compounds such as vitamin E decrease the risk of cardiovascular disease, there has been conflicting evidence of its efficacy when administered alone, along with probable toxicity from high levels of supplementation [14, 15]. The water and lipid-soluble antioxidant, *α*-lipoic acid, decreased reactive oxygen species both at the cell surface and in the mitochondria, increasing uptake of glucose through increasing insulin sensitivity at key muscular sites as well as recycling vitamin E [16, 17]. Treatment with *α*-lipoic acid improved endothelial dysfunction, reduced oxidative stress, improved plasma lipid profiles, and improved insulin sensitivity in high fat-fed Goto-Kakizaki diabetic rats [18]. The combination of vitamin E and *α*-lipoic acid as a regenerative antioxidant protocol improved cardiac performance in aged rats [19]. 

This study has determined whether treatment with a regenerative antioxidant protocol of vitamin E (either *α*-tocopherol or a tocotrienol-rich fraction) together with *α*-lipoic acid can prevent or reverse the cardiovascular and metabolic changes observed with chronic fructose feeding in rats.

## 2. Materials and Methods

### 2.1. Rats

Male Wistar rats (8–10 weeks old weighing 349  ±  8 g; *n*  =  60**)** were obtained from The University of Queensland Biological Resources. The animals were housed in separate cages at the Animal House Facility of the School of Biomedical Sciences, The University of Queensland. Rats were given *ad libitum* access to specific food diets and water and were housed in a 12-hour light/dark environment. All experimental protocols were approved by the Animal Experimentation Ethics Committee of The University of Queensland following the guidelines of the National Health and Medical Research Council of Australia.

### 2.2. Experimental Groups

Rats were fed a diet consisting of fructose (610 g), skim milk powder (200 g), wheat bran (96 g), peanut oil (50 g**)**, Hubbell, Mendel, and Wakeman salt mixture (35 g), and L-methionine (7 g) per kilogram of food [2, 3, 6]. For the control group, fructose was substituted with corn starch (610 g) [2, 3, 6]. Rats were divided into 6 experimental groups. These groups were (i) corn starch (CS) (*n*  =  10), (ii) fructose (F) (*n*  =  10), (iii) fructose + *α*-lipoic acid/*α*-tocopherol prevention (FTPP) (*n*  =  10), (iv) fructose + *α*-lipoic acid/*α*-tocopherol reversal (FTPR) (*n*  =  10), (v) fructose + *α*-lipoic acid/tocotrienol prevention (FTTP) (*n*  =  10), and (vi) fructose + *α*-lipoic acid/tocotrienol reversal (FTTR) (*n*  =  10). Diets were administered to the rats for 16 weeks to allow both induction of significant metabolic and cardiovascular changes as characterized in our previous study [6] and possible reversal of these diet-induced changes for the final 8 weeks of the protocol [2, 3]. The prevention protocols received *α*-lipoic acid with either *α*-tocopherol (group (iii)) or tocotrienol-rich fraction (group (v)) for the full 16 weeks of the diet; the reversal protocols (groups (iv) and (vi)) received these supplements for 8 weeks starting after 8 weeks of the fructose diet. Supplemented diets contained *α*-lipoic acid (1.6 g/kg food) and either *α*-tocopherol (0.84 g/kg food) or tocotrienol-rich fraction (1.56 g/kg food contributing 0.84 g/kg food of a mixture of *α*-tocopherol and tocotrienols).

### 2.3. Assessment of Physiological Parameters

Body weight and food and water intakes were measured daily. Systolic blood pressure was measured after 0, 4, 8, 12, and 16 weeks under light sedation with i.p. injection of Zoletil (tiletamine 15 mg/kg, zolazepam 15 mg/kg), using an MLT1010 Piezo-Electric Pulse Transducer (ADInstruments) and inflatable tail-cuff connected to an MLT844 Physiological Pressure Transducer (ADInstruments) and PowerLab data acquisition unit (ADInstruments, Sydney, Australia). Rats were killed with an intraperitoneal (i.p.) injection of pentobarbitone sodium (100 mg/kg). Blood was taken from the abdominal aorta and centrifuged; the plasma was collected and frozen. Plasma malondialdehyde concentrations were determined by HPLC [20].

### 2.4. Oral Glucose Tolerance Test

Testing was performed after 0, 4, 8, 12, and 16 weeks of diet. After 12 hours of fasting, blood glucose concentrations were measured in blood samples taken from the tail vein. Subsequently, each rat was treated with glucose (2 g/kg) via oral gavage. Tail vein blood samples were taken every 30 minutes up to 120 minutes following glucose administration. The blood glucose concentrations were analyzed with a Medisense Precision Q.I.D glucose meter (Abbott Laboratories, Bedford, USA).

### 2.5. Isolated Heart Preparation

The left ventricular function of the rats in all treatment groups was assessed using the Langendorff heart preparation. Terminal anesthesia was induced via i.p. injection of pentobarbitone sodium (100 mg/kg). Once anesthesia was achieved, heparin (1000 IU) was injected into the right femoral vein. After removal of the heart, isovolumetric ventricular function was measured by inserting a latex balloon catheter into the left ventricle connected to a Capto SP844 MLT844 physiological pressure transducer and Chart software on a Maclab system. All left ventricular end-diastolic pressure values were measured by pacing the heart at 250 beats per minute using an electrical stimulator. End-diastolic pressures were obtained starting from 0 mmHg up to 30 mmHg. The right and left ventricles were separated and weighed. Diastolic stiffness constant (*κ*, dimensionless) was calculated as in previous studies [21, 22]. The liver, kidneys, and spleen were removed and blotted dry for weighing. Organ weights were normalized relative to the tibial length (mg/mm).

### 2.6. Confocal Microscopy

Collagen distribution was measured in the left ventricle following staining with picrosirius red and analyzed by laser confocal microscopy. Tissues were initially fixed for 3 days in Telly's fixative (100 mL of 70% ethanol, 5 mL of glacial acetic acid, and 10 mL of 40% formaldehyde) and transferred into modified Bouin's fluid (85 mL of saturated picric acid, 5 mL of glacial acetic acid, and 10 mL of 40% formaldehyde) for 2 days. The samples were then dehydrated and embedded in paraffin wax. Thick sections (15 *μ*m) were cut and stained and image analysis under the confocal laser scanning microscope was performed as previously described [20, 22].

### 2.7. Statistical Analysis

All data sets were represented as mean ± standard error of mean (SEM). Comparisons of findings between groups were made via statistical analysis of data sets using either an unpaired *t*-test or one-way/two-way analysis of variance (ANOVA) with Bonferroni's multiple-comparison test. A *P* value of <  .05 was considered as statistically significant.

### 2.8. Drugs

*α*-Lipoic acid and *α*-tocopheryl acid succinate were obtained from Associate Professor Jeff Coombes at the School of Human Movement Studies, The University of Queensland. The tocotrienol-rich fraction (Gold Tri.E 70—liquid) was provided by Golden Hope Bioganic, Selangor, Malaysia, part of the Sime Darby group. The product contained *α*-tocotrienol (31.9%), *β*-tocotrienol (2.1%), *γ*-tocotrienol (24.8%), and *δ*-tocotrienol (18.3%) together with *α*-tocopherol (22.9%).

## 3. Results

### 3.1. Physiological and Metabolic Changes

Body weight did not vary among fructose-fed male Wistar rats at the end of the 16-week study protocol although chronic fructose feeding increased left ventricular, liver, and kidney wet weights (Table 1). Addition of the antioxidant treatments to the fructose diet for 16 weeks prevented the increase in left ventricular (LV) and septum weight in comparison to age-matched fructose-fed rats. Addition of the antioxidant treatments to corn starch-fed rats for 8 or 16 weeks did not alter any measured parameter (data not shown). 

**Figure 1:**
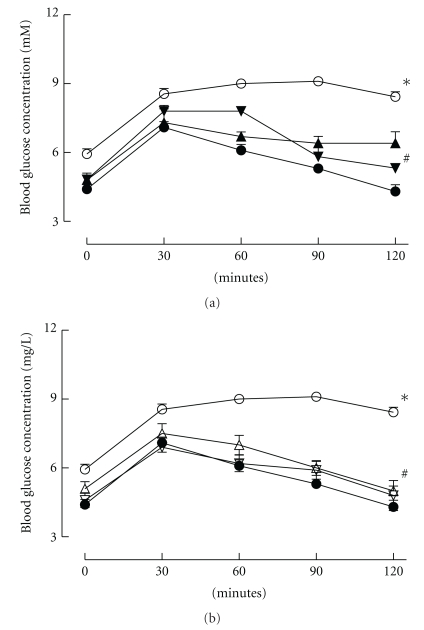
(a): Plasma glucose concentrations following oral gavage of glucose (2 g/kg) recorded after 16 weeks for rats fed with corn starch (*⬤*), fructose (*⚪*), or fructose with *α*-tocopherol and *α*-lipoic acid as either prevention (FTPP) (▲) or reversal (FTPR) (▾) protocols. *P < .05 versus corn starch-fed rats. (b) Plasma glucose concentrations following oral gavage of glucose (2 g/kg) recorded after 16 weeks for rats fed with corn starch (*⬤*), fructose (*⚪*), or fructose with tocotrienol-rich fraction and *α*-lipoic acid as either prevention (FTTP) (*▵*) or reversal (FTTR) (*▿*) protocols. *P < .05 versus corn starch-fed rats; ^#^ *P* < .05 versus fructose-fed rats.

There was an approximate doubling in fasting plasma glucose concentration for the 16-week fructose-fed group in comparison to age-matched controls. After 8 or 16 weeks of the fructose-containing diet, the plasma glucose concentrations measured 120 minutes after oral glucose loading were increased compared to the control group (Figure 1; Table 1). In contrast, the prevention group had plasma glucose concentrations similar to control rats throughout the oral glucose tolerance testing after 8 or 16 weeks of the dietary intervention. The reversal group showed an increased plasma glucose concentration 120 minutes after glucose administration at week 8 (before antioxidant treatment had begun) but normalization of the corresponding blood glucose concentrations after 8-week antioxidant treatment at 16 weeks (Figure 1).

### 3.2. Cardiovascular and Oxidant Changes

**Figure 2:**
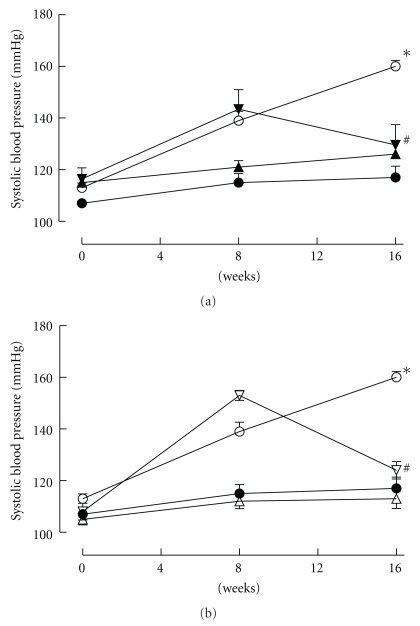
(a): Tail-cuff measurement of systolic blood pressure recorded at 0, 8, and 16 weeks for rats fed with corn starch (*⬤*), fructose (*⚪*), or fructose with *α*-tocopherol and *α*-lipoic acid as either prevention (FTPP) (▲) or reversal (FTPR) (▾) protocols. *P < .05 versus corn starch-fed rats. (b) Tail-cuff measurement of systolic blood pressure recorded at 0, 8, and 16 weeks for rats fed with corn starch (*⬤*), fructose (*⚪*), or fructose with tocotrienol-rich fraction and *α*-lipoic acid as either prevention (FTTP) (*▵*) or reversal (FTTR) (*▿*) protocols. *P < .05 versus corn starch-fed rats; ^#^ *P* < .05 versus fructose-fed rats.

Systolic blood pressure increased in rats with the fructose diet; treatment with antioxidants from the start of the diet prevented this increase (Figure 2). A reversal protocol with the antioxidant treatment starting at week 8 produced a normalization of systolic blood pressure within 4 weeks that was maintained until the end of the study protocol (Figure 2). 

**Figure 3:**
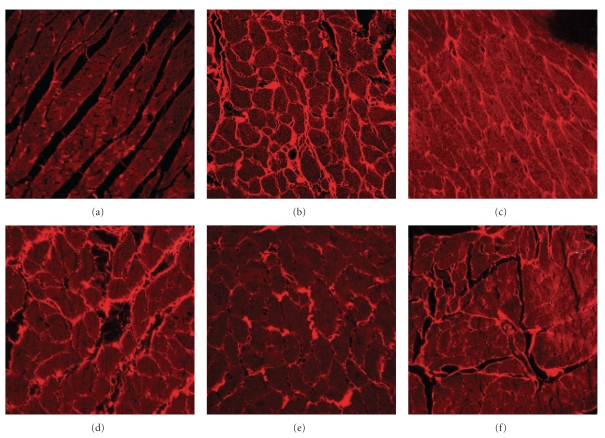
Representative pictures of left ventricular interstitial collagen deposition in rats fed for 16 weeks with corn starch (a), fructose (b), fructose with *α*-tocopherol and *α*-lipoic acid as either prevention (FTPP) (c) or reversal (FTPR) (d) protocols, or fructose with tocotrienol-rich fraction and *α*-lipoic acid as either prevention (FTTP) (e) or reversal (FTTR) (f) protocols.

Fructose feeding increased interstitial collagen deposition in the left ventricle (Table 1). Both prevention and reversal protocols of antioxidant treatment attenuated interstitial collagen deposition (Table 1; Figure 3). Passive diastolic stiffness was increased in the fructose-fed group after 8 and 16 weeks in comparison to age-matched controls (Table 1). Antioxidant treatment as a prevention protocol prevented this increase in diastolic stiffness; further, rats treated as a reversal protocol showed decreased diastolic stiffness compared to fructose-fed rats at 16 weeks that was not different from control rats or rats on the prevention protocol (Table 1). 

Plasma malondialdehyde concentrations were increased in fructose-fed rats. Rats treated with vitamin E and *α*-lipoic acid, either as prevention or reversal protocols, showed plasma malondialdehyde concentrations that did not differ from control rats (Table 1).

## 4. Discussion

### 4.1. Diabetes

Administration of a fructose-rich diet (60%) induces type 2 diabetic complications such as hyperglycemia and hypertension and changes in the structure of target organs such as the heart with moderate cardiac dysfunction [5, 6, 8, 9, 13]. The cardiovascular changes include left ventricular hypertrophy and excess collagen deposition within the interstitium of the heart leading to decreased myocardial function [6, 23]. Many of the complications of chronic fructose intake are due to its rapid metabolism by the liver, causing changes in carbohydrate and lipid metabolism [4, 24]. These chronic changes lead to the development of decreased glucose tolerance, insulin resistance, hyperinsulinemia, and hypertriglyceridemia, major determinants in the formation of type 2 diabetic complications [3, 23, 25]. Hyperglycemia initiates an increase in mitochondrial formation of reactive oxygen species and, as a consequence, oxidative stress [10]. This process has been linked to a downregulation of insulin cell signaling, resulting in insulin resistance [26, 27]. Activation of increased reactive oxygen species production and subsequent oxidative stress therefore plays an integral role in the development and progression of diabetes. Previous studies with antioxidant therapy such as L-arginine, N-acetylcysteine, and Avemar have demonstrated improvement in rat models of type 2 diabetes [8, 28, 29]. Many studies have shown that other natural products may also prevent some of the metabolic changes in diabetes [30–36]. Our study has extended these results by showing that the regenerative antioxidant protocol of the naturally occurring compounds, *α*-lipoic acid and vitamin E, prevented or reversed the diet-induced diabetic complications.

### 4.2. Antioxidants

*α*-Lipoic acid is soluble in both lipid and aqueous cellular phases [16]. Therefore, in lipid phases, *α*-lipoic acid is effective at reducing reactive oxygen species and lipid peroxides in cellular membranes. Furthermore, *α*-lipoic acid has increased access to the cytosol of cells since it is water-soluble and effectively scavenges reactive oxygen species at their mitochondrial source [17]. *α*-Lipoic acid is reduced *in vivo* to dihydrolipoic acid, which is biologically active with its regenerative actions on vitamin E [17]. In humans, *α*-lipoic acid is synthesized in the liver, where it acts as a cofactor for pyruvate dehydrogenase and *α*-ketoglutarate dehydrogenase. These two enzymes, in particular pyruvate dehydrogenase, are found in the mitochondria where they catalyze the oxidative decarboxylation of pyruvate to acetyl-coA necessary in oxidative glucose metabolism [33]. Hence, *α*-lipoic acid has been implicated as a crucial component in the conversion of plasma glucose to energy in mitochondria [16, 37]. In experimental animals, *α*-lipoic acid provided protection against oxidative stress damage to the insulin-secreting *β*-cells of the pancreas, increased glucose metabolism and insulin sensitivity at key muscular sites, and decreased oxidative AGEs and systolic blood pressure [23, 38]. 

Vitamin E inhibits the progression of lipid peroxidation via neutralizing reactive oxygen species at the cellular membrane, thus maintaining the functional and structural integrity of organs such as the cardiovascular system [16, 37]. Following the neutralization of reactive oxygen species, vitamin E is converted to a radical, which no longer possesses any antioxidant properties. In the presence of dihydrolipoic acid, this radical can be converted back to vitamin E, allowing for vitamin E to be distributed through both dietary and regenerative means. Thus, the regenerative process on dietary vitamin E provides a continuous cycle of protection to the cellular structures of key organ sites [19, 38]. In this study, we used two derivatives from the vitamin E family, *α*-tocopherol and a tocotrienol-rich mixture of isomers together with *α*-tocopherol, showing similar responses.

### 4.3. Myocardial Remodeling

Many of the cardiac changes that occur in type 2 diabetes arise from diabetic cardiomyopathy, defined as systolic dysfunction and abnormalities in left ventricular relaxation, usually seen as the first stage of disease progression [39]. In the current study, the isolated Langendorff heart preparation showed an increase in diastolic stiffness, suggesting diastolic dysfunction and a stiffer left ventricle with chronic fructose feeding. These changes could be due to the increased interstitial collagen deposition induced by the hyperglycemic state. This has been previously reported in diabetic rats, where excess collagen deposition was observed in the perivascular and interstitial areas of the myocardium [6, 29]. Increases in collagen deposition are linked to the excess production of advanced glycation end-products (AGEs), as products of nonenzymatic glycosylation in hyperglycemic states [10]. This results in excess collagen cross-linking and deposition, contributing to the increased myocardial stiffness and decreased cardiac function. Cardiovascular remodeling occurs in both type 1 (streptozotocin-diabetic rat) and type 2 (OLETF and high fructose) diabetic models [6, 21, 40].

### 4.4. Hypertension

Increases in systolic blood pressure have been demonstrated in both animal and human studies following the induction of type 2 diabetes [25, 41]. Many diets of Western society are dense in carbohydrates, in particular the monosaccharide fructose [1, 42]. Therefore, using fructose as a diet for rodents has allowed greater understanding of the proposed mechanisms of cardiovascular dysfunction associated with type II diabetes. Fructose feeding increased plasma glucose concentrations and induced glucose intolerance in this study, which is consistent with other studies [6, 29, 31, 34]. Fructose feeding leads to altered glucose metabolism, which results in the shunting of glucose through the polyol pathway, producing increased amounts of fructose and excess aldehyde intermediates such as glyceraldehydes [5]. Excess plasma aldehydes play an essential role in increasing blood pressure by binding sulfhydryl groups of membrane proteins causing increased peripheral vascular resistance [43]. In type 2 diabetes, there is a reduction in the plasma concentrations of glutathione, a reservoir for the aldehyde-binding compound cysteine [44]. Cysteine reacts with aldehydes, breaking down the compound to structures that are easily excreted in the bile and urine [45]. The ability of *α*-lipoic acid and vitamin E to regenerate glutathione allows plasma concentrations to be replenished [16, 17]. *α*-Lipoic acid and vitamin E also reduce the production of excess aldehydes via increasing insulin sensitivity and reducing blood glucose and further by preventing the process of lipid peroxidation, thereby reducing blood pressure [46, 47]. Further, increased reactive free radicals due to fructose feeding may also induce an increase in blood pressure [48, 49]. Studies in humans and animal models suggest a modest reduction in increased blood pressure with dietary antioxidants [48–50] with comparable responses to synthetic antihypertensive compounds such as captopril and metoprolol [51, 52].

In summary, this study indicates that dietary supplements using a regenerative antioxidant protocol such as *α*-lipoic acid and vitamin E can prevent and reverse the metabolic and cardiovascular changes in diet-induced type 2 diabetes.

## Figures and Tables

**Table 1:** Physiological parameters of rats fed with corn starch, fructose, and fructose either with *α*-tocopherol and *α*-lipoic acid as prevention (FTPP) or reversal (FTPR) protocols or with tocotrienol-rich fraction and *α*-lipoic acid either as prevention (FTTP) or reversal (FTTR) protocols. ^a^Values for rats fed the fructose diet for 8 weeks were taken from [6]. Values are mean ± SEM; number of experiments in parentheses. LV: left ventricle; RV: right ventricle; *P < .05 versus corn starch-fed rats; ^#^ *P* < .05 versus fructose-fed rats.

Parameter	Corn starch (16 weeks)	Fructose (8 weeks)^a^	Fructose (16 weeks)	FTPP (16 weeks)	FTPR (16 weeks)	FTTP (16 weeks)	FTTR (16 weeks)
Body weight at 0 weeks (g)	339 ± 8 (*n* = 10)	351 ± 10 (*n* = 10)	349 ± 5 (*n* = 10)	347 ± 10 (*n* = 10)	334 ± 5 (*n* = 10)	356 ± 5 (*n* = 10)	352 ± 6 (*n* = 10)
Body weight at 16 weeks (g)	533 ± 14 (*n* = 10)	459 ± 13 (*n* = 9)	521 ± 8 (*n* = 10)	502 ± 12 (*n* = 10)	510 ± 9 (*n* = 10)	493 ± 12 (*n* = 10)	502 ± 8 (*n* = 9)
Fasting plasma glucose concentrations (mmol/L)	2.8 ± 0.2 (*n* = 6)	6.1 ± 0.1* (*n* = 6)	5.9 ± 0.2* (*n* = 6)	4.8 ± 0.2^#^ (*n* = 6)	4.7 ± 0.1^#^ (*n* = 6)	4.1 ± 0.3^#^ (*n* = 6)	4.3 ± 0.4*^#^* (*n* = 6)
Plasma glucose concentration (mmol/L) (after 120-minute glucose loading)	5.9 ± 0.1 (*n* = 6)	8.0 ± 0.2* (*n* = 8)	9.1 ± 0.2* (*n* = 6)	6.4 ± 0.5^#^ (*n* = 6)	5.3 ± 0.1^#^ (*n* = 6)	5.0 ± 0.2^#^ (*n* = 6)	4.8 ± 0.7^#^ (*n* = 6)
LV—interstitial collagen (% of total area)	4.6 ± 0.7 (*n* = 3)	N/A	18.7 ± 2.7* (*n* = 3)	12.6 ± 1.8^#^ (*n* = 3)	13.7 ± 2.3^#^ (*n* = 3)	10.8 ± 2.9^#^ (*n* = 3)	11.2 ± 1.5^#^ (*n* = 3)
Diastolic stiffness constant (*κ*)	20.5 ± 0.4 (*n* = 6)	23.9 ± 1.4* (*n* = 6)	25.8 ± 1.1* (*n* = 6)	21.1 ± 0.6^#^ (*n* = 6)	21.9 ± 0.5^#^ (*n* = 6)	17.7 ± 1.2^#^ (*n* = 6)	19.8 ± 2.0^#^ (*n* = 6)
LV + septum (mg/g body wt)	1.7 ± 0.09 (*n* = 6)	2.10 ± 0.07* (*n* = 8)	2.05 ± 0.1* (*n* = 6)	1.8 ± 0.05^#^ (*n* = 6)	2.1 ± 0.2* (*n* = 6)	1.9 ± 0.1^#^ (*n* = 6)	2.0 ± 0.1* (*n* = 6)
RV (mg/g body wt)	0.53 ± 0.03 (*n* = 6)	0.55 ± 0.03* (*n* = 11)	0.47 ± 0.02 (*n* = 6)	0.47 ± 0.02 (*n* = 6)	0.50 ± 0.05 (*n* = 6)	0.4 ± 0.1 (*n* = 6)	0.4 ± 0.1 (*n* = 6)
Liver (mg/g body wt)	26.7 ± 0.6 (*n* = 6)	36.7 ± 1.5* (*n* = 8)	39.6 ± 3.6* (*n* = 6)	29.4 ± 1.5^#^ (*n* = 6)	31.6 ± 0.8^#^ (*n* = 6)	33.3 ± 2.5* (*n* = 6)	35.2 ± 1.4* (*n* = 6)
Spleen (mg/g body wt)	2.0 ± 0.1 (*n* = 6)	2.1 ± 0.1 (*n* = 9)	2.4 ± 0.2 (*n* = 6)	2.0 ± 0.2 (*n* = 6)	2.2 ± 0.1 (*n* = 6)	1.9 ± 0.1 (*n* = 6)	2.0 ± 0.2 (*n* = 6)
Kidneys (mg/g body wt)	5.15 ± 0.1 (*n* = 6)	7.31 ± 0.2* (*n* = 8)	6.30 ± 0.4* (*n* = 6)	6.0 ± 0.2* (*n* = 6)	6.0 ± 0.3* (*n* = 6)	7.0 ± 0.3* (*n* = 6)	6.3 ± 0.2* (*n* = 6)
Plasma malondialdehyde concentration (*μ*mol/L)	26.9 ± 0.7 (*n* = 6)	42.8 ± 1.9* (*n* = 3)	33.9 ± 1.0* (*n* = 6)	27.9 ± 2.0^#^ (*n* = 6)	27.6 ± 1.6^#^ (*n* = 6)	26.4 ± 1.5^#^ (*n* = 6)	26.8 ± 2.3^#^ (*n* = 6)
